# Natural Product Erianin Inhibits Bladder Cancer Cell Growth by Inducing Ferroptosis via NRF2 Inactivation

**DOI:** 10.3389/fphar.2021.775506

**Published:** 2021-10-29

**Authors:** Yu Xiang, Xiaying Chen, Wengang Wang, Lijuan Zhai, Xueni Sun, Jiao Feng, Ting Duan, Mingming Zhang, Ting Pan, Lili Yan, Ting Jin, Quan Gao, Chengyong Wen, Weirui Ma, Wencheng Liu, Deqiang Wang, Qibiao Wu, Tian Xie, Xinbing Sui

**Affiliations:** ^1^ School of Pharmacy and Department of Medical Oncology, The Affiliated Hospital of Hangzhou Normal University, Hangzhou Normal University, Hangzhou, China; ^2^ Key Laboratory of Elemene Class Anti-Cancer Chinese Medicines, Engineering Laboratory of Development and Application of Traditional Chinese Medicines, Collaborative Innovation Center of Traditional Chinese Medicines of Zhejiang Province, Hangzhou Normal University, Hangzhou, China; ^3^ College of Pharmacy, Chengdu University of Traditional Chinese Medicine, Chengdu, China; ^4^ Zhejiang Chinese Medical University, Hangzhou, China; ^5^ Department of Medical Oncology, Affiliated Hospital of Jiangsu University, Jiangsu University, Zhenjiang, China; ^6^ State Key Laboratory of Quality Research in Chinese Medicines, Faculty of Chinese Medicine, Macau University of Science and Technology, Macau, China

**Keywords:** Erianin, ferroptosis, bladder cancer, nuclear factor E2-related factor 2, natural product

## Abstract

Erianin, a natural product derived from *Dendrobium chrysotoxum Lindl*, has been proved to play antitumor activity in various cancers. However, the effects and molecular mechanisms of erianin in bladder cancer cells remain unexplored. In this study, we found that erianin triggered cell death and cell cycle arrest in bladder cancer cells. Then we demonstrated that erianin could promote the accumulation of lethal lipid-based reactive oxygen species (ROS) and the depletion of glutathione (GSH), suggesting the induction of ferroptosis. In the further study, the ferroptosis inhibitor deferoxamine (DFO), N-Acetylcysteine (NAC) and GSH but not necrostatin-1, CQ or Z-VAD-FMK rescued erianin-caused cell death, showing ferroptosis played a major role in erianin-caused cell death. *In vivo*, we also showed that erianin suppressed the tumor growth by inducing ferroptosis. Mechanistically, we demonstrated that nuclear factor E2-related factor 2 (NRF2) inactivation was a key determinant of ferroptosis caused by erianin. In bladder cancer cells, the compound tert-butylhydro-quinone (TBHQ), an activator of NRF2, suppressed erianin-induced ferroptosis. Whereas, NRF2 inhibition used shRNA augmented the ferroptosis response induced by erianin treatment. In conclusion, our data provide the first evidence that erianin can initiate ferroptosis-like cell death and lipid peroxidation in bladder cancer, which will hopefully become a promising anticancer compound for the treatment of bladder cancer.

## Introduction

Ferroptosis is a newly defined form of regulated cell death, which is characterized by iron-dependent peroxidation of the lipid membrane induced by reactive oxygen species (ROS) (Yu et al., 2020). Increasing evidence has depicted an essential role for ferroptosis as either the cause or consequence for the pathophysiological process of many human diseases, including acute lung injury (Li et al., 2020), intervertebral disc degeneration (Zhang et al., 2020), ulcerative colitis (Wang et al., 2020), and so on. Recently, more and more studies have provided intriguing insights into the interplay between ferroptosis and cancer because ferroptosis could eliminate cancer cells with an apoptosis-independent manner (Badgley et al., 2020; Lee et al., 2020). In consequence, ferroptosis has attracted more and more attention and will hopefully offer a novel strategy for cancer treatment.

It’s our knowledge that a lot of anticancer drugs come from natural sources, including naturally occurring forms and synthetic derivatives. Recently, increasing evidence show that several natural compounds can either induce or inhibit ferroptosis, further expanding their therapeutic potentials. Erianin, extracted from rare traditional Chinese medicine *Dendrobium chrysotoxum Lindl*, was a small-molecule natural compound with a wide range of anticancer potential. Erianin has been reported to inhibit colorectal cancer cells growth by downregulating the transcriptional activity of β-catenin (Sun et al., 2020), induce apoptosis in nasopharyngeal carcinoma by decreasing the phosphorylation of ERK1/2 (Liu et al., 2019), and suppress bladder cancer cell growth through JNK pathways and the mitochondrial apoptosis (Zhu et al., 2004). In our previous study, erianin inhibited the growth and migration via inducing Ca/CaM-dependent ferroptosis and inhibiting cell migration in lung cancer cells (Chen et al., 2020a). However, the underlying molecular mechanism and the anticancer effect of erianin are not well exploited in bladder cancer.

In this study, we focused the effect of erianin on the viability in two different bladder cancer cells KU-19–19 and RT4. The result showed that erianin exerted its anticancer potential through inducing cell death and G2/M-phase arrest in a dose-dependent manner. Then for the first time we showed that ferroptosis facilitated erianin-induced cell death of bladder cancer *in vitro* and *in vivo*, that was confirmed by accompanied with ROS accumulation, GSH depletion, and lipid peroxidation. Moreover, erianin-induced cell death could be rescue by co-treatment with ROS inhibitor N-acetyl-l-cysteine (NAC) or glutathione (GSH) in bladder cancer cells. Mechanistically, we demonstrated that nuclear factor E2-related factor 2 (NRF2) inactivation was a key determinant of erianin-caused ferroptosis. In bladder cancer cells, the compound tert-butylhydro-quinone (TBHQ), an activator of NRF2, suppressed erianin-induced ferroptosis. Whereas, NRF2 inhibition by shRNA augmented the ferroptosis response induced by erianin treatment. In conclusion, our data for the first time demonstrated that erianin suppressed bladder cancer cell growth by inducing ferroptosis via inhibition of NRF2 signaling pathway.

## Materials and Methods

### Cell Culture

RT4 and KU-19–19 cell lines were acquired from ATCC (American Type Culture Collection, Manassas, VA, United States). RT4 and KU-19–19 were cultivated in McCoy’s 5A Medium Modified and 1640 Medium Modified comprising 10% fetal bovine serum and double antibiotic, requiring the temperature at 37°C with the routine cultivated conduction (5% CO_2_, 95% air).

### Reagents and Antibodies

Purified erianin (>98%) (Cat: B20844) was gained from Shanghai Yuanye Biological Co., Ltd. All antibody follow-up experiments used were anti-xCT (Cat: ab175186), anti-Glutaminase (Cat: ab93424), anti-GPX4 (Cat: ab125066), anti-Heme Oxygenase-1 (Cat: ab189491) were from Abcam (Cambridge, United Kingdom), anti-FTH1 (Cat: 4393S), anti-NRF2 (Cat:12721S), anti-GAPDH (Cat: 5174S) were provided by Cell Signaling Technology (Danvers, United States). And inhibitors mentioned in experiments were deferoxamine (Cat: S5742) (Selleck, Houston, United States), chloroquine (Cat: C6628) (Sigma-Aldrich, St. Louis, United States), Z-VAD-FMK (Cat: HY-16658B), N-Acetylcysteine (Cat: HY-B0215), necrostatin-1 (Cat: HY-15760), L-Glutathione reduced (Cat: HY-D0187), TBHQ (Cat:HY-100489) were obtained from MedChem Express (New Jersey, United States), pLVX-U6-NRF2-shRNA1-PGK-EGFP-E2A-Puro (Cat:p24452) (miaolingbio, Wuhan, China).

### Cell Viability Assay

The CCK-8 (Cat: MA0218) (meilunbio, Dalian, China) proliferation assay was used to examine cell viability of KU-19–19 and RT4 after different treatments. About of 5 × 10^3^ cells/well were added into a 96-well plate. And then erianin or inhibitors were used with diverse concentrations for 24 h. After treating for 24 h, CCK-8 solution 10 μl/100 μl to a well was added and placed for 2 h at 37°C before the absorbance at a test wave length of 450 nm.

### Apoptosis Assays

Following the manufacturer’s instruction, the percentage of cell death used the Annexin V: FITC Apoptosis Detection kit (Cat: 556547) (BD, San Jose, United States). Approximately 6 × 10^5^ cells/well were added in 6-well plate and erianin was given to treatment. The cells to be tested were collected in 100 μl 1×binding buffer placed in an ice box, and stained with FITC Annexin V and PI at room temperature out of Sun for 15 min. Then 200 μl 1×binding buffer was added. Cells were processed and analyzed with a FACS Calibur Flow Cytometer (CytoFLEX S) (Beckman Coulter, United States).

### Cell Cycle Staining Assay

The cell cycle was made according to the requirement of cell cycle staining kit (Cat: CCS012) (Multi Science, Hangzhou, China). Cells were adherent and incubated with erianin in 6-well plate for 24 h. Cells collected after treatments were dyed with PI and analyzed by Flow Cytometer.

### Measurement of Reactive Oxygen species

Approximately 6×10^5^ cells/well were grown in 6-well plate. Cells were treated differently for 24 h after attachment, then 10 μM H2DCF-DA (Cat: S0033) (Beyotime Biotechnology, Shanghai, China) was added to incubate for 1 h. Rinsing the cells twice with PBS to remove residual H2DCF-DA. After that, the labeled cells were trypsinized and 200 μl PBS was added. H2DCF-DA is used to staining and detect ROS generation. The results were processed and analyzed by the FACS. Each condition was demanded to collect minimum of 10,000 cells.

### Measurement of Glutathione

Approximately 6×10^5^ cells/well were placed into 6-well plate. After attached, KU-19–19 and RT4 were treated as described previously for 24 h. Protein concentration was determined with BCA assay kit (Cat: P0010S) (Beyotime Biotechnology, Shanghai, China). Following the manufacturer’s instruction, total glutathione was measured as instruction of GSH assay kit (Cat: A006-2-1) (Nanjing Jiancheng, Nanjing, China).

### Malondialdehyde Assay

Cells were plated and incubated with erianin in 6-well plate for 24 h. Following the manufacturer’s instructions, malondialdehyde was detected of MDA assay kit (Cat: S0131S) (Beyotime Biotechnology, Shanghai, China) based on the protein concentration.

### Measurement of Ferrous Ion

Approximately 6×10^5^ cells/well were placed into 6-well plate. Following the manufacturer’s instructions, ferrous ion was detected of Phen Green SK (Cat: P14313) (Thermo Scientific, Rockford, United States). The results were processed and analyzed by the FACS. Each condition was demanded to collect minimum of 10,000 cells.

### Western Blot Analysis

Approximately 3×10^6^ cells were placed into 10 cm dish. After attached, KU-19–19 and RT4 were dealt with differently for 24 h. And the cells were collected and lysed for 30 min on ice. Then collecting the supernatant after centrifugation at 10^4^ rpm/min, then the concentration of protein was measured by BCA kit. Protein was dissolved in SDS-PAGE (12%), and electro-transferred to PVDF membranes. The Membranes were blocked, washed, antibody incubation, and enhanced chemiluminescence detection.

### *In vivo* Tumor Model

The experimental mice were permited by the Animals Use and Care Committee at Hangzhou Normal university (No. 2021–1112). 4-weeks-old female BALB/c nude mice aged were injected into 5×10^5^ cells. Every 2 days mice weight and tumor size were assessed, and the tumor volume (V) was calculated with the formula: (maximum length) × (maximum width)^2^/2. Once tumors were found, the mice were stochastically divided into 2 groups: the control (PBS) group and the treatment (erianin 100 mg/kg) group. For 14 days, mice were injected intraperitoneally with drugs once a day, then puting the mice to death, after that tumors were taken for IHC (immunohistochemical) analysis.

### Statistical Analysis

The results were expressed as mean ± SD. The significance of the statistical results used the *t*-test of GraphPad Prism. Unless otherwise indicated, all studies are conducted at least three times.

## Results

### Erianin Inhibited Cell Proliferation and Triggered Cell Death in Bladder Cancer Cells

**FIGURE 1:**
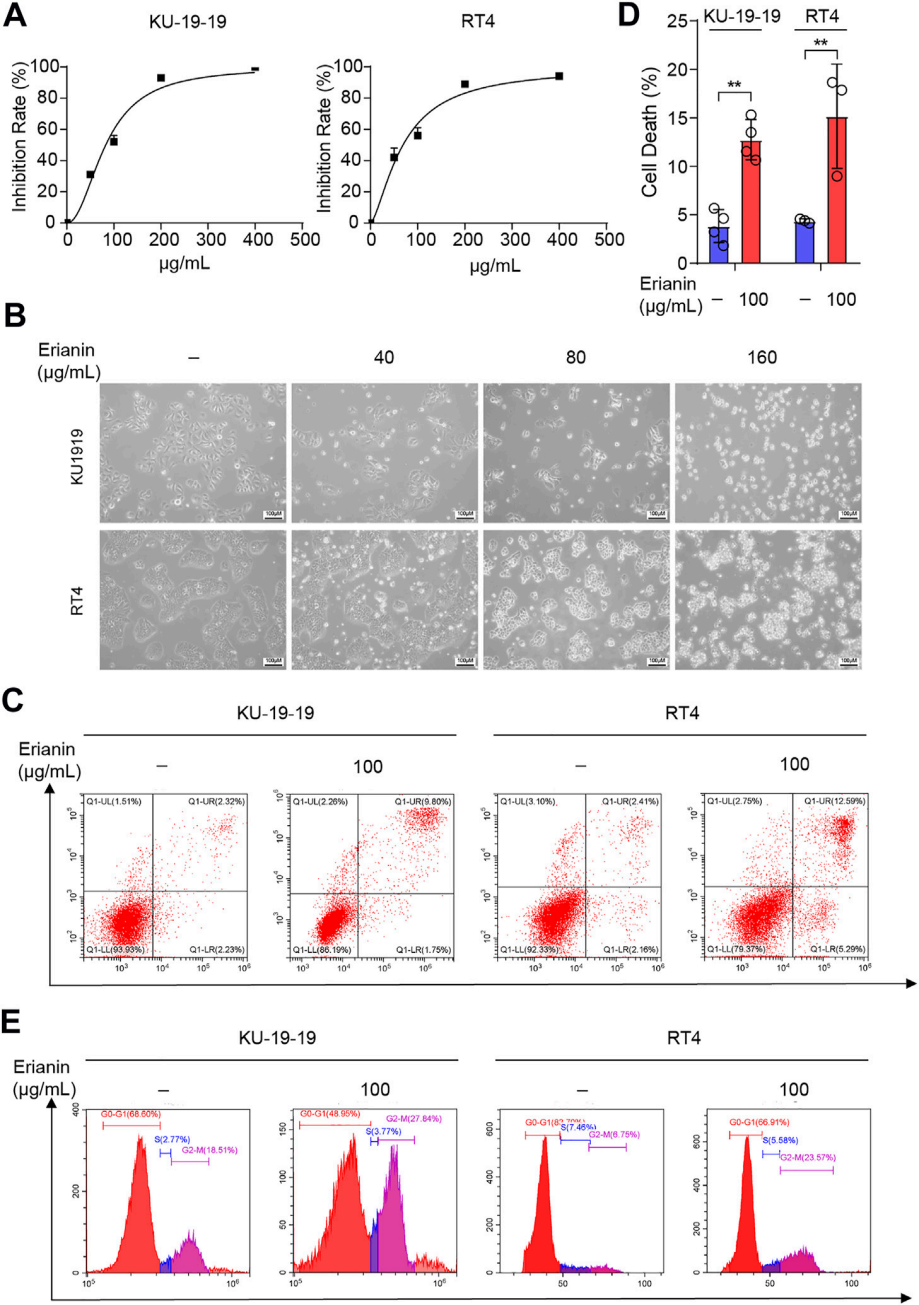
Erianin inhibited cell proliferation and triggered cell death in bladder cancer cells. **(A)** Cell proliferation of KU-19–19 and RT4 was assessed by CCK-8 assay after the treatment with different dose of erianin treatment for 24 h. **(B)** The cell morphology was observed by microscope. **(C,D)** Flow cytometry analysis of cell death by Annexin V-FITC/PI staining in KU-19–19 and RT4 cells were treated with erianin or DMSO control for 24 h, and the quantification of percentage of the cell death was shown. *p < 0.05,**p < 0.01. **(E)** The percentage of cells treated with erianin in each phase was assessed by flow cytometry.

In order to explore the antitumor potential of erianin in bladder cancer, erianin was treated with different concentrations for 24 h in KU-19–19 and RT4 cells, and the cell growth potential was evaluated by CCK-8 assay. As a result, erianin treatment decreased cell viability in a dose-dependent (Figures 1A,B) and time-dependent (Supplementary Figure S1A,B) manner. In order to determine whether erianin could inhibit the growth of bladder cancer cells by inducing the cell death, we performed Annexin V-FITC/PI staining and analyzed by flow cytometry. In Figures 1C,D, a markedly high percentage of the dead cells was discovered in bladder cancer cells after the treatment with erianin. Then flow cytometry was performed to confirm whether erianin inhibited the cell proliferation through inducing cell cycle arrest. The result revealed erianin treatment significantly improved the proportion in G2/M phase (Figure 1D).

### Ferroptosis Predominantly Contributed to Cell Death Inducing by Erianin in Bladder Cancer

**FIGURE 2:**
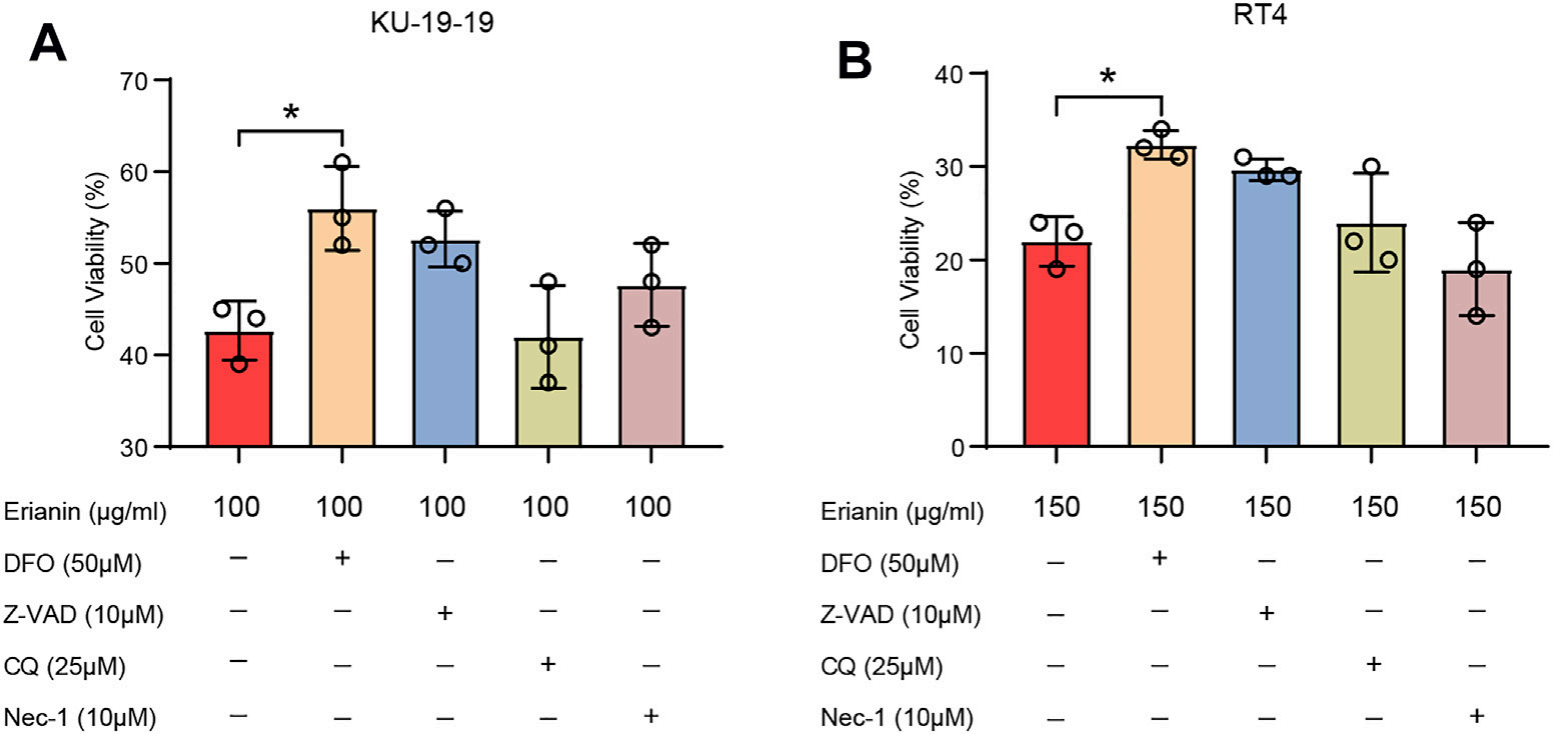
KU-19–19 and RT4 were treated with erianin in combination with or without other cell death inhibitors Nec-1, DFO, Z-VAD and CQ for 24 h, then cell viability was assayed. *p < 0.05.

The inhibitors of several different signaling pathway were utilized to confirm which cell death program mainly caused the cell death induced by erianin. The results showed that Z-VAD-FMK (inhibitor of pan-caspase), necrostatin-1 (Nec-1, inhibitor of necroptosis) or chloroquine (CQ, inhibitor of autophagy) could not reverse cell death in KU-19–19 and RT4 after the treatment with erianin. Whereas, iron chelator deferoxamine mesylate (DFO) remarkably reduced erianin-induced cell death, indicating ferroptosis is the dominated method generated erianin-caused cell death (Figures 2A,B).

**FIGURE 3:**
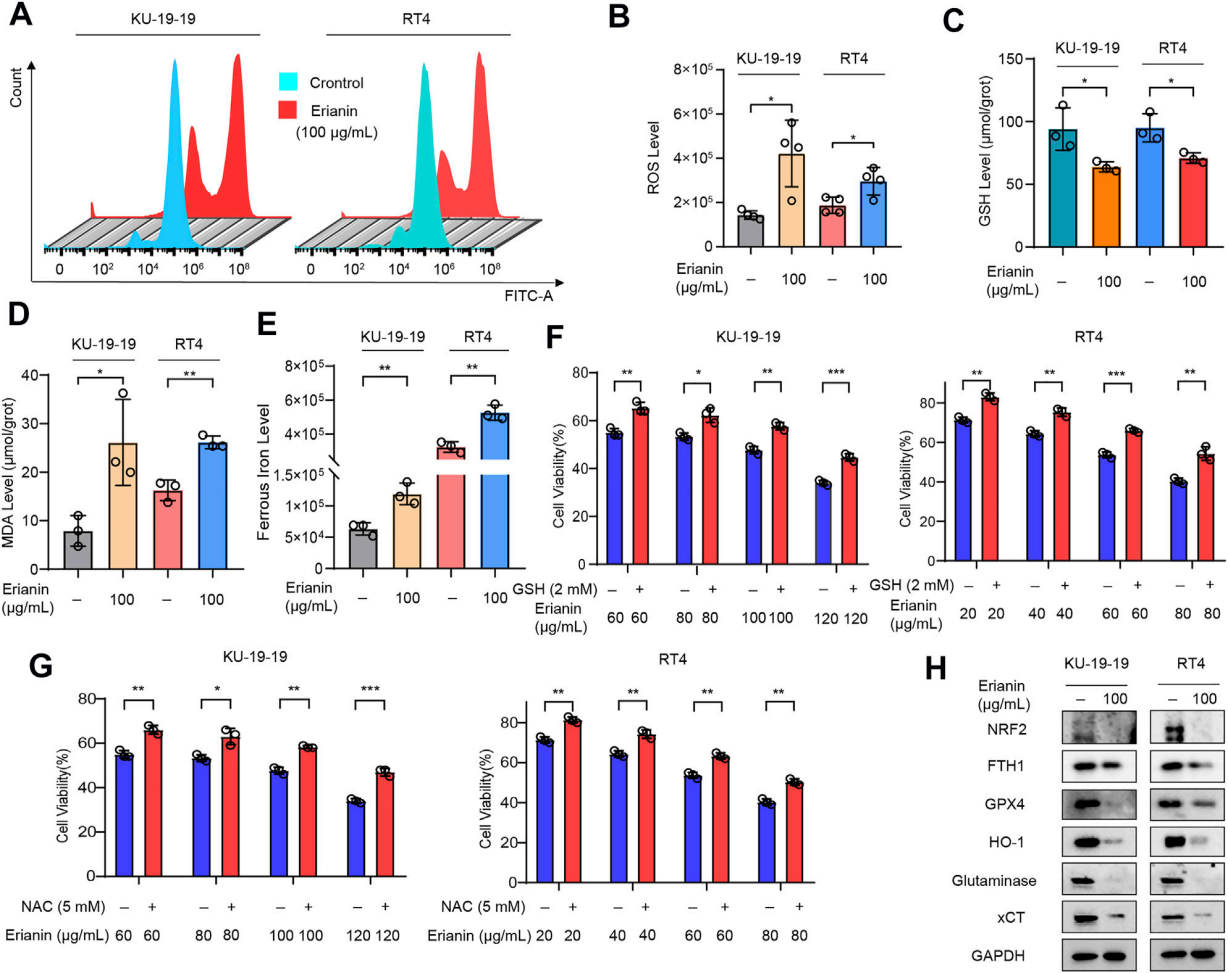
Erianin triggered ferroptosis in bladder cancer cells. **(A,B)** KU-19–19 and RT4 cells were treated with erianin for 24 h and ROS level was analyzed by a flow cytometer, the mean ± s.d. is shown. *p < 0.05. **(C)** The GSH level in KU-19–19 and RT4 cells was detected after the treatment with erianin for 24 h, *p < 0.05. **(D)** The MDA level in KU-19–19 and RT4 cells was detected after the treatment with erianin for 24 h, *p < 0.05,**p < 0.01. **(E)** The iron level in KU-19–19 and RT4 cells was detected after the treatment with erianin for 24 h, **p < 0.01. **(F)** KU-19–19 and RT4 cells were treated with erianin with or without the oxygen-derived free radicals scavenger GSH for 24 h, and cell viability was assayed, **p < 0.01, ***p < 0.001. **(G**) KU-19–19 and RT4 cells were treated with erianin with or without the ROS scavenger NAC for 24 h, and cell viability was assayed, **p < 0.01, ***p < 0.001. **(H**) Several ferroptosis related proteins were determined by western blotting.

It is well known that lipid peroxidation and GSH depletion are important phenomena in ferroptosis. Thus, the level of intracellular ROS, GSH and malondialdehyde (MDA) in KU-19–19 and RT4 cells were further detected following treatment with erianin. The results showed that the increased level of ROS (Figures 3A,B) and the decreased level of GSH (Figure 3C) were observed after erianin treatment. Meanwhile, erianin treatment obviously upregulated the MDA level (Figure 3D) and triggered ferrous iron accumulation (Figure 3E) in these bladder cancer cells. Furthermore, the anticancer activity of erianin could be dramatically rescued by co-treatment with oxygen-derived free radicals scavenger GSH (Figure 3F) and ROS scavenger N-ace-tylcysteine (NAC) (Figure 3G).

Subsequently, the effect of erianin on several ferroptotic proteins was detected by western blotting. As shown in Figure 3H, the protein levels of nuclear factor E2-related factor 2 (NRF2), ferritin heavy chain 1 (FTH1), glutathione peroxidase 4 (GPX4), heme oxygenase 1 (HO-1), glutaminase (GLS) and solute carrier family 7 member 11 (xCT/SLC7A11) were significantly decreased in KU-19–19 and RT4 cells after erianin treatment compared with the control group (Figure 3G). In conclusion, these results suggested that ferroptosis predominantly contributed to erianin-caused cell death in bladder cancer cells.

### Erianin Exerted Antitumor Effect by Inducing Ferroptosis *in vivo*

**FIGURE 4:**
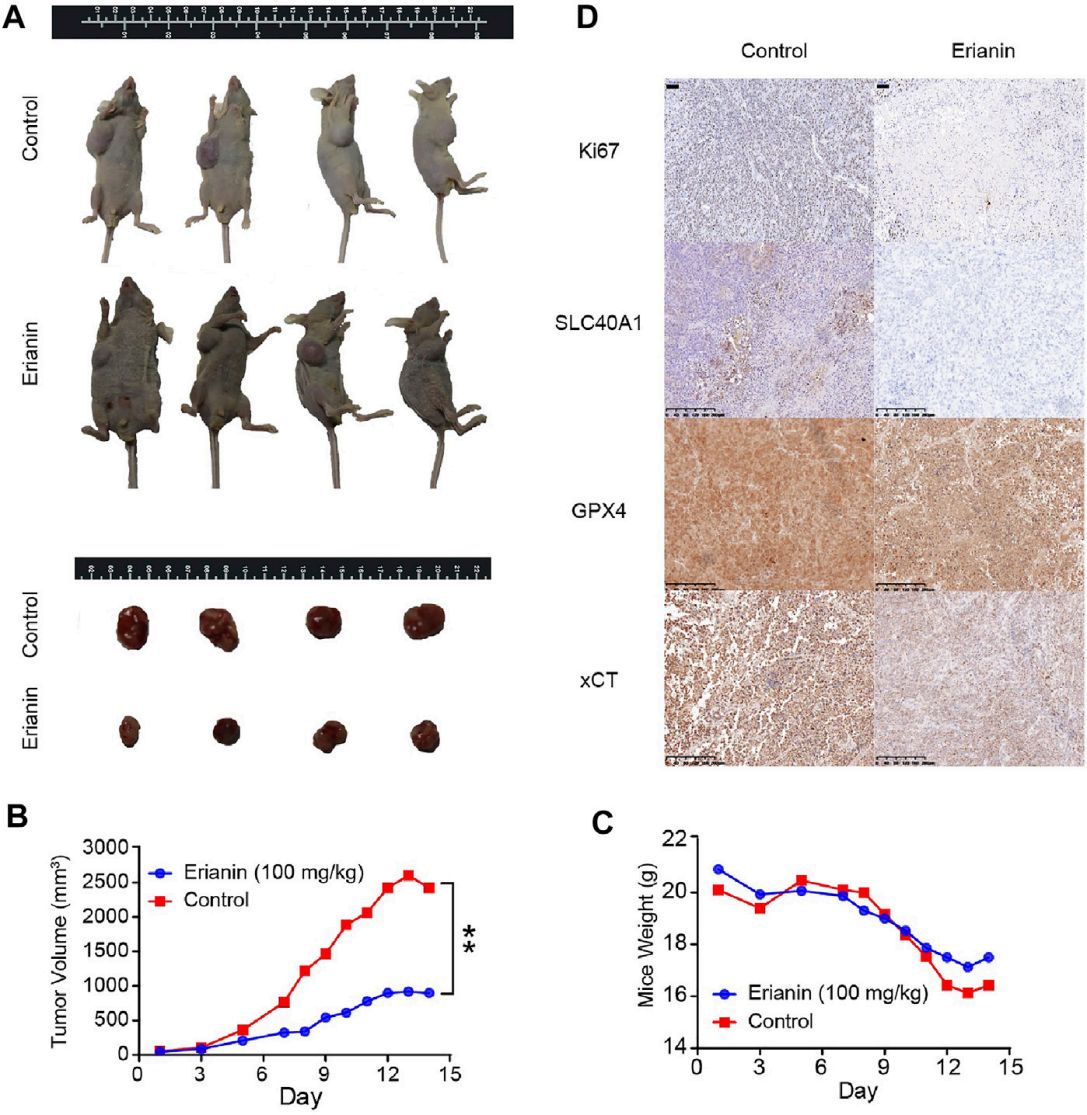
Erianin suppressed tumor growth by inducing ferroptosis *in vivo*. **(A)** Representative image of mice and xenograft tumors at the endpoint of experiment. **(B)** The average tumor volume from different groups. Values are shown as mean ± SD, **p < 0.01. **(C)** The average mice weight from different groups. Values are shown as mean ± SD. **(D)** The expression of Ki67, GPX4, SLC40A1 and xCT were detected by IHC.

Based on the above findings, we considered whether erianin administration could contribute to ferroptosis-like cell death in a subcutaneous xenograft tumor model. Therefore, BALB/c nude mice with KU-19–19 subcutaneous xenografts were stochasticly divided into two experimental groups: the control group (DMSO) and the erianin treatment group (100 mg/kg). Our data revealed that the growth of KU-19–19 xenograft tumors was dramatically reduced upon erianin treatment (Figures 4A,B). However, the body weight between the vehicle control group and erianin-treated group was no statistically significant difference (Figure 4C).

We next investigated the expression of Ki67 and several ferroptotic proteins by immunohistochemical (IHC). Ki67 staining showed that erianin treatment suppressed Ki67 expression in tumor tissues (Figure 4D), suggesting erianin could suppress the growth of bladder tumor. Also, the protein levels of SLC40A1, GPX4 and xCT were obviously decreased in tumors from erianin treatment group (Figure 4D). These results collectively support the idea that erianin suppressed tumor growth by inducing ferroptosis *in vivo*.

### Erianin Triggered Ferroptosis via the Nuclear Factor E2-Related Factor 2 Pathway

**FIGURE 5:**
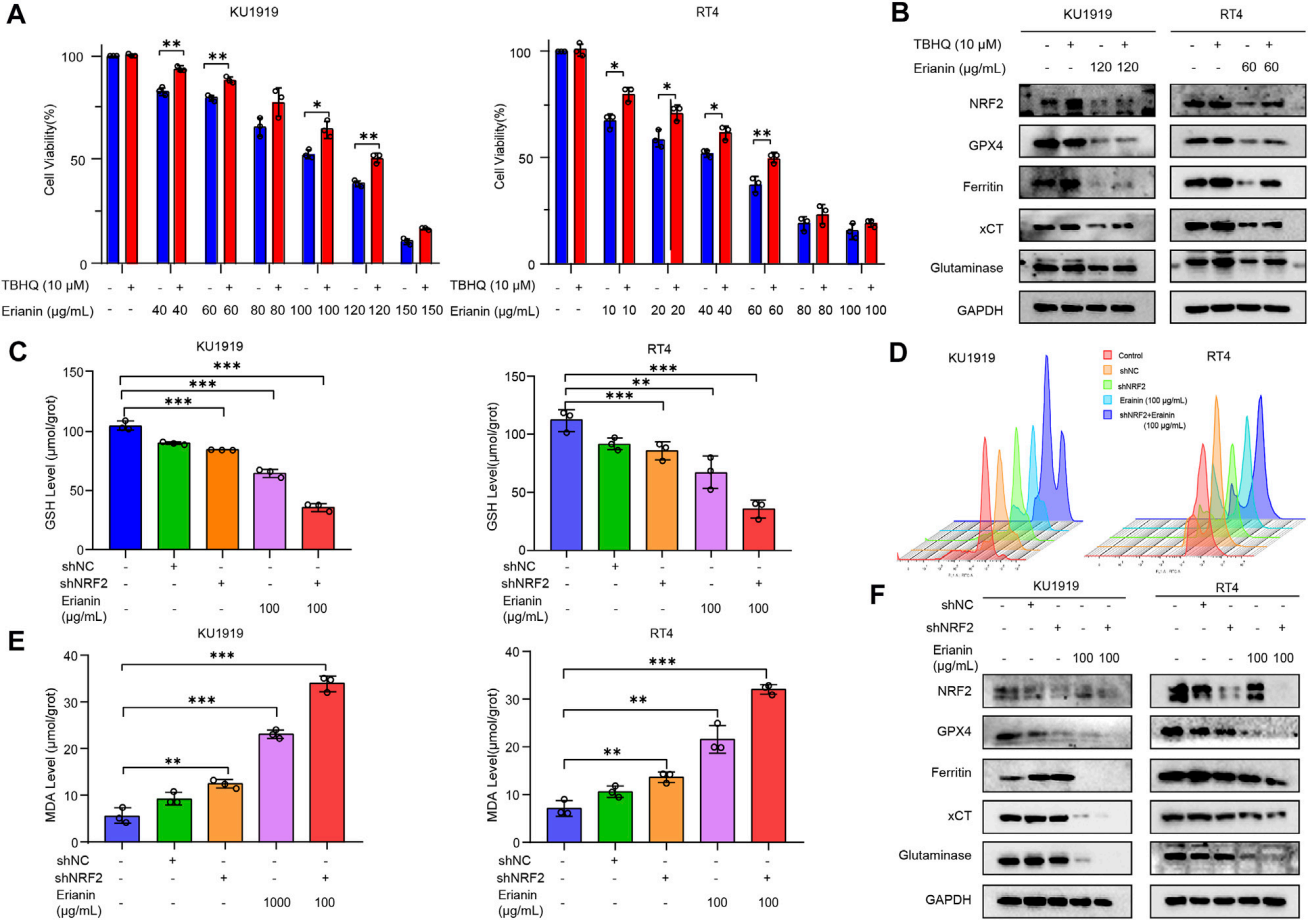
Erianin triggered ferroptosis via the NRF2 pathway. **(A)** Cell viability was quantified by CCK-8 assay in KU-19–19 and RT4 cells treated with the combination of erianin and TBHQ. *p < 0.05,**p < 0.01. **(B)** The expression of NRF2, GPX4, ferritin, xCT and glutaminase was analyzed in KU-19–19 and RT4 cells treated with the combination of erianin and TBHQ. **(C)** The GSH level in KU-19–19 and RT4 cells was detected after the treatment with erianin and shNRF2, **p < 0.01,***p < 0.001. **(D)** The ROS level in KU-19–19 and RT4 cells was detected after the treatment with erianin and shNRF2, **p < 0.01. **(E)** The MDA level in KU-19–19 and RT4 cells was detected after the treatment with erianin and shNRF2, **p < 0.01,***p < 0.001. **(F)** The expression of NRF2, GPX4, ferritin, xCT and glutaminase was analyzed in KU-19–19 and RT4 cells treated with erianin and shNRF2.

Recent studies have established that NRF2 signaling has a key role in cancer development and ferroptosis inhibition. Therefore, we speculated whether erianin might also induce ferroptosis in bladder cancer through modulation of NRF2. As shown in Figure 3H, the treatment of erianin inhibited the activation of NRF2. In order to further evaluate the relationship between erianin-induced ferroptosis and NRF2 signaling, we specifically enhanced NRF2 activation by using tert-butylhydro-quinone (TBHQ), a pharmacological NRF2 activator. We found that TBHQ treatment (10 μM) protected against erianin-induced cell death in KU-19–19 and RT4 cells (Figure 5A). Moreover, upregulation of GPX4, ferritin, xCT and glutaminase expression is also observed after the co-treatment with erianin and TBHQ (Figure 5B). This result suggests that erianin-mediated NRF2 inhibition probably promotes ferroptosis as a pro-death effect on bladder cancer cells.

Next, we used short hairpin RNA (shRNA) to knockdown NRF2 expression in KU-19–19 and RT4 cells and tested the effect of such inhibition on erianin-triggered ferroptosis. As a result, GSH level were significantly reduced (Figure 5C), but the activity of ROS and MDA were greatly increased after being pretreated with shNRF2 (Figures 5D,E). Meanwhile, the blockage of NRF2 decreased the protein level of GPX4, ferritin, xCT and glutaminase (Figure 5F) and increased the cell death induced by erianin treatment (Figure S1C,D). In general, these results indicated that NRF2 signaling is a key mediator of erianin-triggered ferroptosis in bladder cancer cells.

## Conclusion

Ferroptosis, a new kind of cell death accompanied by iron-dependent lipid peroxidation, has recently been recognized as an important role in degenerative diseases, infection diseases and cancer (Chen et al., 2019; Yu et al., 2019; Chen et al., 2020b). Therefore, activating ferroptosis may be a potential strategy for the treatment of cancer, particularly for eradicating traditional therapies-resistant aggressive malignancies. Recently, great progress has been made in the study of novel anticancer drugs induced by ferroptosis. As a critical sources of novel compounds used for anticancer drugs, natural molecules inducing ferroptosis are attracting increasing interest in cancer.

Erianin, the major bioactive flavones extracted from *Dendrobium chrysotoxum Lindl*, also called as shihu in traditional Chinese medicine. Growing evidence supports that erianin exerts antitumor activity in various cancer types. Erianin could suppressed cell proliferation and migration (Zhang et al., 2019), triggered mitochondrial apoptosis (Zhu et al., 2019), autophagy (Chen YT. et al., 2020) and cell cycle arrest (Chen YT. et al., 2020). However, the function of erianin has not been well explored in bladder cancer.

Here, we first showed that erianin inhibited cell proliferation and triggered cell death in bladder cancer cells. Next, we demonstrated that ferroptosis as the predominant method contributed to erianin-induced cell death of bladder cancer both *in vitro* and *in vivo*, which was confirmed by accompanied with ROS accumulation, GSH depletion, lipid peroxidation and downregulation of FTH1, GPX4, HO-1, GLS and xCT/SLC7A11. Moreover, ROS inhibitor NAC or glutathione GSH could rescue erianin-induced ferroptotic cell death. Mechanistically, we showed NRF2 was a key determinant for erianin-triggered ferroptosis. NRF2 activation by TBHQ treatment protected against erianin-induced cell death and increased the expression of GPX4, ferritin, xCT and glutaminase. On the other hand, NRF2 knockdown shRNA increased the activity of ROS and MDA but decreased GSH level and the expression of negative regulatory proteins for ferroptosis.

In brief, our data indicate that inducing ferroptosis is the main mechanism mediating antitumor activity of erianin, and erianin is expected to serve as a promising compound for the treatment of bladder cancer.

## Data Availability

The raw data supporting the conclusions of this article will be made available by the authors, without undue reservation.

## References

[B1] Badgley M. A. Kremer D. M. Maurer H. C. DelGiorno K. E. Lee H. J. Purohit V. (2020). Cysteine Depletion Induces Pancreatic Tumor Ferroptosis in Mice. Science 368, 85–89. 10.1126/science.aaw9872 32241947 PMC7681911

[B2] Chen P. Li X. Zhang R. Liu S. Xiang Y. Zhang M. (2020b). Combinative Treatment of β-elemene and Cetuximab Is Sensitive to KRAS Mutant Colorectal Cancer Cells by Inducing Ferroptosis and Inhibiting Epithelial-Mesenchymal Transformation. Theranostics 10, 5107–5119. 10.7150/thno.44705 32308771 PMC7163451

[B3] Chen P. Wu Q. Feng J. Yan L. Sun Y. Liu S. (2020a). Erianin, a Novel Dibenzyl Compound in Dendrobium Extract, Inhibits Lung Cancer Cell Growth and Migration via Calcium/calmodulin-dependent Ferroptosis. Signal. Transduct Target. Ther. 5, 51. 10.1038/s41392-020-0149-3 32382060 PMC7205607

[B4] Chen W. Wu G. Zhu Y. Zhang W. Zhang H. Zhou Y. (2019). HOXA10 Deteriorates Gastric Cancer through Activating JAK1/STAT3 Signaling Pathway. Cancer Manag. Res. 11, 6625–6635. 10.2147/CMAR.S201342 31406476 PMC6642621

[B5] Chen Y. T. Hsieh M. J. Chen P. N. Weng C. J. Yang S. F. Lin C. W. (2020c). Erianin Induces Apoptosis and Autophagy in Oral Squamous Cell Carcinoma Cells. Am. J. Chin. Med. 48, 183–200. 10.1142/S0192415X2050010X 31903779

[B6] Gong Y.-Q. Fan Y. Wu D.-Z. Yang H. Hu Z.-B. Wang Z.-T. (2004). *In Vivo* and *In Vitro* Evaluation of Erianin, a Novel Anti-angiogenic Agent. Eur. J. Cancer 40, 1554–1565. 10.1016/j.ejca.2004.01.041 15196540

[B7] Lee H. Zandkarimi F. Zhang Y. Meena J. K. Kim J. Zhuang L. (2020). Energy-stress-mediated AMPK Activation Inhibits Ferroptosis. Nat. Cel Biol. 22, 225–234. 10.1038/s41556-020-0461-8 PMC7008777 32029897

[B8] Li Y. Cao Y. Xiao J. Shang J. Tan Q. Ping F. (2020). Inhibitor of Apoptosis-Stimulating Protein of P53 Inhibits Ferroptosis and Alleviates Intestinal Ischemia/reperfusion-Induced Acute Lung Injury. Cell Death Differ 27, 2635–2650. 10.1038/s41418-020-0528-x 32203170 PMC7429834

[B9] Liu Y. T. Hsieh M. J. Lin J. T. Chen G. Lin C. C. Lo Y. S. (2019). Erianin Induces Cell Apoptosis through ERK Pathway in Human Nasopharyngeal Carcinoma. Biomed. Pharmacother. 111, 262–269. 10.1016/j.biopha.2018.12.081 30590314

[B10] Sun Y. Li G. Zhou Q. Shao D. Lv J. Zhou J. (2020). Dual Targeting of Cell Growth and Phagocytosis by Erianin for Human Colorectal Cancer. Drug Des. Devel Ther. 14, 3301–3313. 10.2147/DDDT.S259006 PMC7429191 32848368

[B11] Wang S. Liu W. Wang J. Bai X. (2020). Curculigoside Inhibits Ferroptosis in Ulcerative Colitis through the Induction of GPX4. Life Sci. 259, 118356. 10.1016/j.lfs.2020.118356 32861798

[B12] Yu B. Choi B. Li W. Kim D. H. (2020). Magnetic Field Boosted Ferroptosis-like Cell Death and Responsive MRI Using Hybrid Vesicles for Cancer Immunotherapy. Nat. Commun. 11, 3637. 10.1038/s41467-020-17380-5 32686685 PMC7371635

[B13] Yu M. Gai C. Li Z. Ding D. Zheng J. Zhang W. (2019). Targeted Exosome-Encapsulated Erastin Induced Ferroptosis in Triple Negative Breast Cancer Cells. Cancer Sci. 110, 3173–3182. 10.1111/cas.14181 31464035 PMC6778638

[B14] Zhang X. Huang Z. Xie Z. Chen Y. Zheng Z. Wei X. (2020). Homocysteine Induces Oxidative Stress and Ferroptosis of Nucleus Pulposus via Enhancing Methylation of GPX4. Free Radic. Biol. Med. 160, 552–565. 10.1016/j.freeradbiomed.2020.08.029 32896601

[B15] Zhang X. Wang Y. Li X. Yang A. Li Z. Wang D. (2019). The Anti-carcinogenesis Properties of Erianin in the Modulation of Oxidative Stress-Mediated Apoptosis and Immune Response in Liver Cancer. Aging (Albany NY) 11, 10284–10300. 10.18632/aging.102456 31754081 PMC6914393

[B16] Zhu Q. Sheng Y. Li W. Wang J. Ma Y. Du B. (2019). Erianin, a Novel Dibenzyl Compound in Dendrobium Extract, Inhibits Bladder Cancer Cell Growth via the Mitochondrial Apoptosis and JNK Pathways. Toxicol. Appl. Pharmacol. 371, 41–54. 10.1016/j.taap.2019.03.027 30946863

