# Supplementary material for: Natural Product Erianin Inhibits Bladder Cancer Cell Growth by Inducing Ferroptosis via NRF2 Inactivation
Source: Front Pharmacol. 2021 Oct 29;12:775506. doi: 10.3389/fphar.2021.775506 (PMC8585785; doi:10.3389/fphar.2021.775506)
Supplement: Supplementary file 1 [file DataSheet1.PDF]

Supplemental Information

Natural product erianin inhibits bladder cancer cell growth by inducing ferroptosis via NRF2 inactivation

Yu Xiang[1, 4, †], Xiaying Chen[1, 4, †], Wengang Wang[3, †], Lijuan Zhai[2], Xueni Sun[1, 4], Jiao Feng[1, 4], Ting Duan[1, 4], Mingming Zhang[1, 4], Ting Pan[1, 4], Lili Yan[1, 4], Ting Jin[1, 4], Quan Gao[1, 4], Chengyong Wen[1, 4], Weirui Ma[1, 4], Wencheng Liu[1, 4], Deqiang Wang[5, *], Qibiao Wu[6*], Tian Xie[1, 3, 4, *], Xinbing Sui[1, 4, 5, 6, *]

†These authors made equal contributions to this work

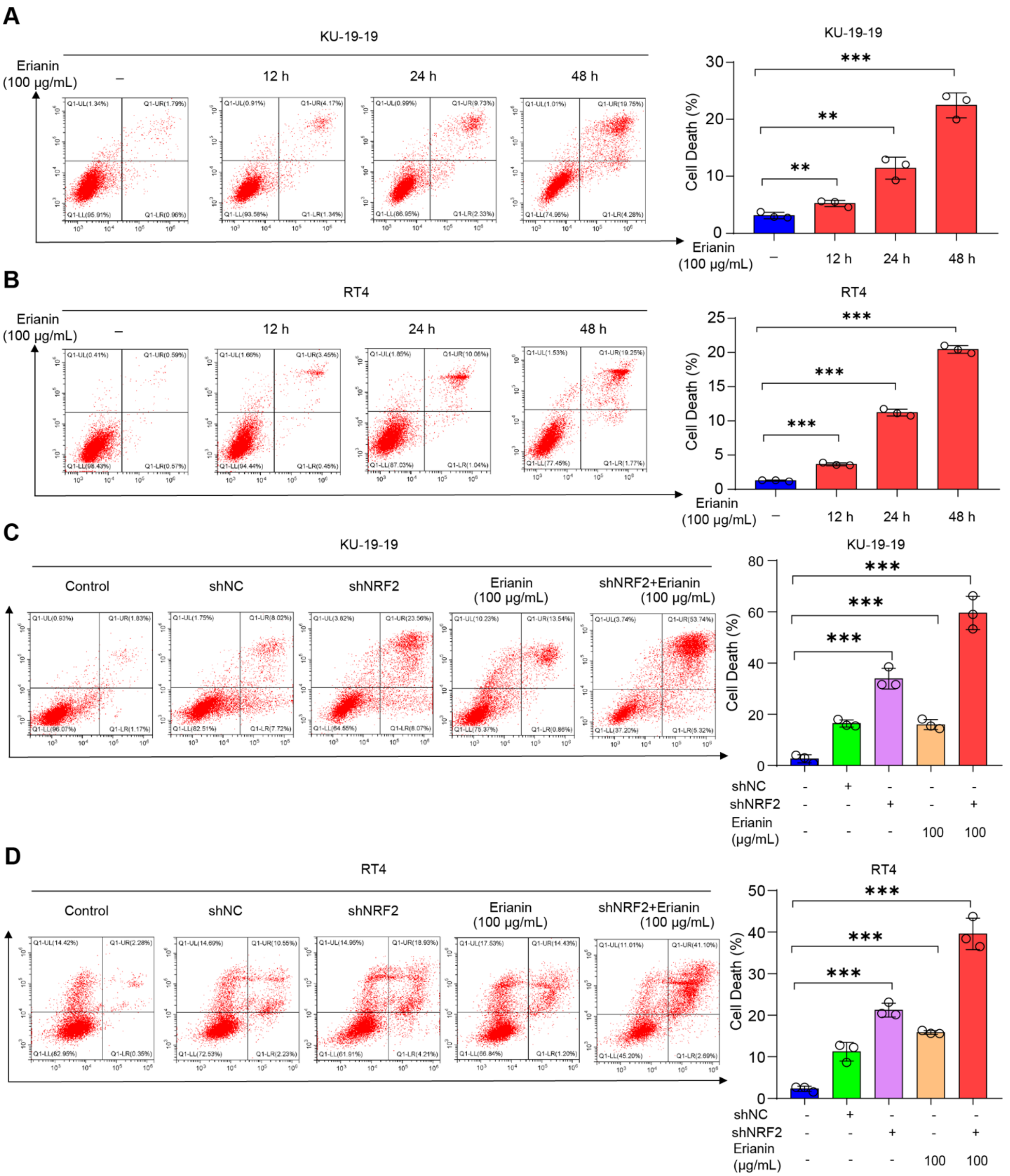

FIGURE S1. (**A-B**) Flow cytometry analysis of cell death by Annexin V-FITC/PI staining in KU-19-19 and RT4 cells were treated with erianin or DMSO control for different time, and the quantification of percentage of the cell death was shown. **$P < 0.01$, ***$P < 0.001$. (**C-D**) The effect of NRF2 knockdown on erianin-triggered cell death. , ***$P < 0.001$.
